# Nutrition screening tools for risk of malnutrition among hospitalized patients: A protocol for systematic review and meta analysis

**DOI:** 10.1097/MD.0000000000022601

**Published:** 2020-10-23

**Authors:** Regina Cortes, Miquel Bennasar-Veny, Enrique Castro-Sanchez, Sergio Fresneda, Joan de Pedro-Gomez, Aina Yañez

**Affiliations:** a Son Espases University Hospital, Balearic Islands Health Service; b Department of Nursing and Physiotherapy, Balearic Islands University; c Research group on Evidence, Lifestyles and Health, Instituto de Investigación Sanitaria de les Illes Balears (IdISBa), Balearic Islands, Palma, Spain; d City, University of London, London, United Kingdom.

**Keywords:** hospitalization, malnutrition, meta-analysis, nutrition assessment, screening, systematic review

## Abstract

**Background:**

Malnutrition is a clinical problem with a high prevalence in hospitalized adult patients. Many nutritional screening tools have been developed but there is no consensus on which 1 is more useful. The purpose of this review protocol is to provide an overview of which nutritional screening tool is most valid to identify malnutritional risk in hospitalized adult patients and to analyze the sensitivity and specificity of the different tools.

**Methods:**

The protocol of this systematic review and meta-analysis was registered on the INPLASY website (https://inplasy.com/inplasy-2020-9-0028/) and INPLASY registration number is INPLASY202090028. We will perform a systematic literature search of main databases: PubMed, EMBASE, CINAHL and Web of Science and the Cochrane database. Also, grey literature will be search. Peer-reviewed studies published in English, Portuguese or Spanish language will be selected. Screening of titles, abstract and full text will be assessed for eligibility by 2 independent blinded reviewers and any discrepancies will be resolved via consensus. After screening the studies, a meta-analysis will be conducted, if it is possible.

**Results:**

Results from this systematic review will help health professionals to identify malnutrition in hospitalized patients and to make decisions to prevent or treat it as well as provide new clues to researchers.

**Conclusion:**

Our systematic review will provide aknowledge about the most valid malnutrition risk screening tool in hospitalized adult patients.

## 1 Introduction

Malnutrition is a condition characterized by a negative balance of energy and/or proteins that leads to altered body composition as a consequence of the decrease in muscle and/or fat mass. Such status leads to diminished physical and mental function and impaired clinical outcomes from disease.^[1]^

Although a universally accepted definition of malnutrition is still lacking,^[2]^ the European Society for Clinical Nutrition and Metabolism (ESPEN)^[3]^ defined malnutrition by the presence of one of the following criteria:

1. body mass index (BMI) < 18.5 kg/m^2^;
2. unintentional weight loss and reduced BMI (age dependent cut-offs) or
3. unintentional weight loss and reduced gender dependent fat free mass index.^[4]^

On the other hand, the American Society for Parenteral and Enteral Nutrition (ASPEN)^[5]^ establishes that at least 2 of the following 6 criteria should be fulfilled to meet the diagnostic criteria of malnutrition: low energy intake, weight loss, loss of muscle mass, loss of subcutaneous fat, fluid accumulation, and diminished hand grip strength.

Malnutrition is a clinical problem of high prevalence, affecting between 30% to 50% of hospitalized patients, depending on age, the screening tool used and the hospital setting.^[6–10]^ Furthermore, malnutrition is associated with increased morbidity and mortality, length of hospital stay and likelihood of hospital readmission, which in turns raises healthcare costs.^[8,11–13]^

The risk of malnutrition in hospitals is associated to other diseases, pharmacological treatments and diagnostic and therapeutic interventions.^[14]^ Many studies agree on the efficiency and effectiveness of the prevention of malnutrition by nutritional status screening and assessment during hospital admission, to adequately provide nutrition therapy when it is necessary.^[15–17]^ However, whilst clinical practice guidelines recommend that nutritional screening should be routinely performed at hospital admission together with nutritional assessment, if indicated, the reality is very different, and malnutrition remains highly prevalent among hospital patients.^[18]^

There is no anthropometric or analytical value alone useful to carry out a diagnosis of malnutrition and there is no international consensus about clinical diagnosis.^[2,19]^ Although a nutritional screening allows for the detection of patients at high risk, such screening is only performed in ∼10% to 20% of hospitalized patients, even in hospitals with a clinical nutrition department.^[6,11]^ Furthermore, only half of hospitalized patients undergo laboratory tests, anamnesis or physical examination to evaluate their nutritional status.^[20]^

Additionally, there is also some confusion in the literature regarding the terminology surrounding malnutrition. For example, nutritional screening (which refers to the identification of malnutritional risk) and nutritional assessment (which aims to establish a nutritional diagnosis to identify malnutrition)^[18]^ are different steps of nutrition care in hospitalized patients.

There are many nutritional screening tools^[21]^ as Nutritional Risk Screening 2002 (NRS-2002),^[22]^ recommended by ESPEN; Malnutrition Universal Screening Tool (MUST),^[23]^ used at community and hospital levels; Mini Nutritional Assessment (MNA),^[24]^ used in patients over 65 years; Short Nutritional Assessment Questionnaire (SNAQ)^[25]^ used, regardless of age, in hospitals, nursing homes and at community level; and Malnutrition Screening Tool (MST)^[26]^ completed by the patient. While numerous nutritional screening tools are in use, their levels of validity, reliability, generalizability and agreement vary.^[27]^ These tools assess different clinical aspects of patients with objective measures (recent weight loss, changes in intake, presence of physical and/or mental illnesses related to a decrease in intake or malabsorption of nutrients) and assign a score that allows classifying patients according to their risk of malnutrition.

Nevertheless, there are screening tools that identify clinical variables similar to the previous ones but that classify the risk of malnutrition according to a subjective final assessment made by the observer, such as the Subjective Global Assessment (SGA),^[28]^ recommended by the ASPEN. This tool, used in all healthcare settings, has been used as a gold standard for the validation of other nutrition screening tools.^[26,29]^

Despite the availability of these nutritional screening tools, there is no international consensus on which is the most valid tool to use in the hospital setting.^[30]^

### 1.1 Research questions

The questions of interest for this systematic review are:

1. Which nutritional screening tool is most valid to identify malnutritional risk in hospitalized adult patients?
2. What are the estimates for sensitivity, specificity, positive predictive value (PPV), negative predictive value (NPV) and likelihood ratios?

### 1.2 Objectives

The aims of this study are:

1. To provide an overview about which nutritional screening tool is most valid to identify malnutritional risk in hospitalized adult patients.
2. To identify the sensitivity, specificity, PPV, NPV and likelihood ratios of different tools.

## 2 Methods

### 2.1 Study protocol and registration

The study protocol has been registered in INPLASY, an International Platform of Registered Systematic Review and Meta-analysis Protocols (https://inplasy.com/inplasy-2020-9-0028/) (Registration No. INPLASY202090028, doi: 10.37766/inplasy2020.9.0028). This protocol is prepared according to the Preferred Reporting Items for Systematic Reviews and Meta-Analyses Protocols (PRISMA-P) statements,^[31,32]^ and the systematic review was equally reported according to PRISMA guidelines.^[31,33]^

### 2.2 Strategy of literature searches

We will search the following databases: PubMed, EMBASE, CINAHL (via the EBSCO), Web of Science and the Cochrane database. Peer-reviewed studies published in English, Portuguese or Spanish language will be selected. Search terms will include controlled terms from MeSH in PubMed, EMtree in EMBASE and CINAHL headings in CINAHL as well as free text terms. The key search terms that will be combined include “nutrition assessment”, “nutritional screening tool”, “malnutrition screening”, “malnutrition”, “adult”, and “hospital” (Table 1). Reference lists will also be verified for relevant citations. The search strategy will be performed in cooperation with a research librarian and it is presented in online supplementary additional file.

**Table 1:**
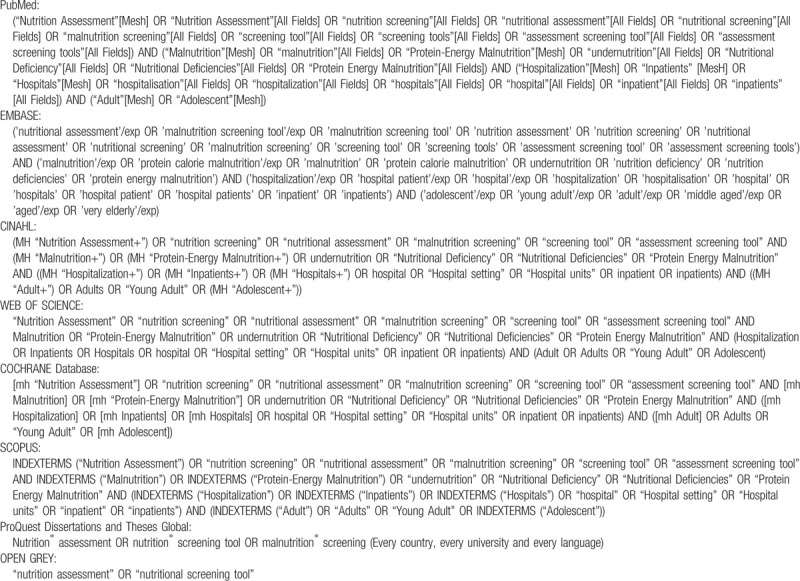
Databases search strategy.

#### 2.2.1 Additional search strategy (identification of grey literature)

Unpublished literature will be identified through the Information System on Gray Literature in Europe (Open Gray), Conference Proceedings of the Web of Science and ProQuest Dissertations and Theses Global. If necessary, the authors will be contacted to obtain a full report of the findings, if available. Data from conference proceedings will not be included in the review due to the limited information available to carry out the methodological quality assessment.

### 2.3 Eligibility criteria and exclusion criteria

#### 2.3.1 Eligibility criteria

The inclusion criteria will be the following:

1. validation studies of nutritional screening tools developed to identify malnutrition or risk of malnutrition;
2. studies focused on hospitalized adults (18 to 85 years old).

#### 2.3.2 Exclusion criteria

We will exclude studies focused on:

1. residents in nursing homes or long-term facilities care facilities;
2. children and young adults (up to 18-years of age);
3. pregnant women;
4. terminal or palliative patients;
5. patients with eating disorders;
6. nutrition indexes (NRI, GNRI, etc.) instead of screening tools;
7. reporting in languages other than English, Portuguese or Spanish language.

### 2.4 Data collection and analyses

#### 2.4.1 Selection of studies

**Figure 1:**
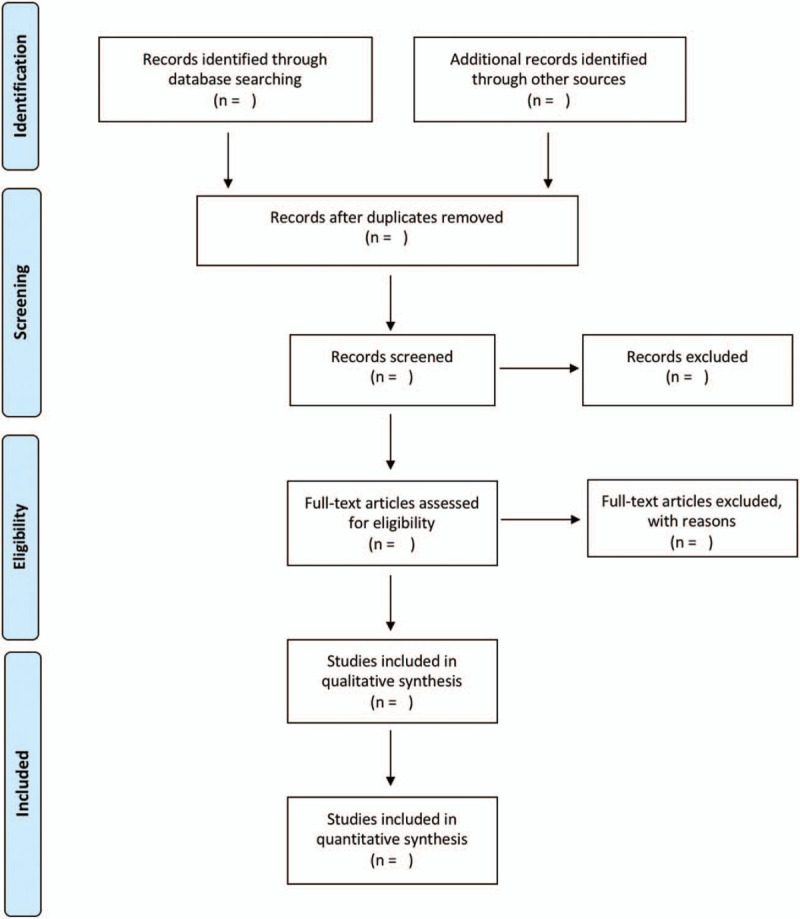
Study screening process: Preferred Reporting Items for Systematic Reviews and Meta-Analyses flow diagram.

References of the studies identified by the literature search strategy will be imported into EndNote X9 (Clarivate analytics, Philadelphia, USA) literature management software, and duplicates will be removed. To ensure the quality of the process, 2 blinded reviewers will separately screen the study titles and abstracts for relevance. Should disagreements arise between the reviewers, then the full text of the document will be retrieved. Disagreements will be resolved via consensus; the opinion of a third reviewer will be sought as necessary. After this initial selection, all potentially eligible references will be evaluated to see if they meet the inclusion criteria. The reviewers will contact the authors to obtain full versions of the articles that cannot be obtained in full text. To reduce the risk of bias, a pilot exercise will be carried out to apply the inclusion criteria in a sample of 20 references. A summary of the study screening process will be presented using the PRISMA flow chart (Fig. 1). Excluded studies will be listed in a table with the reasons for their exclusion.

#### 2.4.2 Data extraction and management

The characteristics of the studies and study data will be managed using Microsoft Excel 2019 (Microsoft Corp, Redmond, WA, www.microsoft.com) and Review Manager software (RevMan version 5.3, Copenhagen, Denmark: The Nordic Cochrane Centre, the Cochrane Collaboration 2014), respectively. Three reviewers (RC, SF and JDP) will separately collect data including eligible studies characteristics (e.g., name of first author, publication year, country, journal title, study design, participants, sample size, study setting, risk assessment tools), outcomes (sensitivity, specificity, and likelihood ratios) and declarations of interests. Where possible, these outcomes will be calculated if they are not already reported within the study. Missing or incomplete data will be obtained by contacting authors of the studies directly.

#### 2.4.3 Quality evaluation

In order to assess the methodological quality of each included studies, we will use the Quality Assessment of Diagnostic Accuracy Studies (QUADAS-2) checklist as a critical appraisal instrument (http://www.bris.ac.uk/quadas).^[34]^

Disagreements will be resolved by having a discussion or consultations with another reviewer (MBV).

A concordance analysis among reviewers will be carried out during the review process and this will be subsequently incorporated into the results using Cohen's kappa coefficient. The following labels will be assigned to the corresponding ranges of kappa: poor (<0.00), slight (0.00–0.20), fair (0.21–0.40), moderate (0.41–0.60), substantial (0.61–0.80) and almost perfect (0.81–1.00).^[35]^

#### 2.4.4 Data synthesis and meta-analysis

Relevant data checked and agreed by 3 reviewers will be exported from Microsoft Excel to RevMan 5.3 and STATA version 16.1 (Stata, College Station, TX) for quantitative synthesis. A *P* value <.05 will be considered statistically significant for all analyses.

A narrative synthesis of the outcomes will be presented in the final review. Meta-analysis of sensitivity, specify, PPV, NPV and likelihood ratios (LHs) will be performed for each nutrition screening tool.

#### 2.4.5 Assessment of heterogeneity

Statistical heterogeneity will be quantified by the Cochranes *Q* statistic and the inconsistency index (*I*^2^) test.^[36]^ The heterogeneity will be stratified into tree levels: 25% low heterogeneity, 25% to 50% moderate heterogeneity and > 50% high heterogeneity.^[37]^ We will use fixed-effects model with weighting of the studies if there is no evidence of significant heterogeneity, and will use random-effects model with weighting of the studies when there is heterogeneity between studies.

An assessment of homogeneity in terms of methodology and outcomes will be performed. Should there be high heterogeneity, then only a narrative synthesis will be performed instead, without meta-analysis.

We will use forest plots to graphically represent sensitives, specificities, PPV, NPV and LHs with 95% confidence intervals (CIs) for each nutrition screening tool. We will also conduct sensitivity analysis to assess the influence of each study on the overall effect by removing one of the studies in each round and publication bias across studies will be evaluated using funnel plots and Eggers test.^[38]^

### 2.5 Ethical principles and publication

Ethical approval will not be sought as this is a protocol for a systematic review. Patient and public were not involved in this study. The findings of the study will be disseminated through international and national conferences, and in a peer-reviewed journals. The results will also be communicated to patients and patient representatives in suitable language via popular science publications and on institutional websites.

## 3 Discussion

Nutritional screening should be routinely performed at hospital admission, with the goal of decreasing malnutrition related morbimortality; however, there remains a lack of consensus on which tool is best for determining malnutritional risk on clinical practice.

This systematic review aims to summarise the evidence on the validity of different nutritional screening tools. Results of this systematic review will therefore provide new insights into malnutrition prevention and treatment to promote new clinical practice recommendations and polices. The systematic review may also highlight limitations or gaps in the evidence for future research.

May be interesting to explore results depending on gender, as well as high income and low-income settings. Equally, would be interesting to think about clinician-administered or patient-administered tools.

## Author contributions

**Conceptualization:** Miquel Bennasar-Veny, Aina Yañez.

**Data curation:** Regina Cortes, Sergio Fresneda.

**Investigation:** Miquel Bennasar-Veny, Enrique Castro-Sanchez, Aina Yañez.

**Methodology:** Miquel Bennasar-Veny.

**Supervision:** Joan De Pedro-Gómez.

**Validation:** Miquel Bennasar-Veny, Enrique Castro-Sanchez.

**Visualization:** Regina Cortes.

**Writing – original draft:** Regina Cortes, Miquel Bennasar-Veny.

**Writing – review & editing:** Enrique Castro-Sanchez, Sergio Fresneda, Joan De Pedro-Gómez, Aina Yañez.

## References

[1] Moriana M Civera M Artero A Validity of subjective global assessment as a screening method for hospital malnutrition. Prevalence of malnutrition in a tertiary hospital. Endocrinol Nutr 2014;61:184–9. 24342427 10.1016/j.endonu.2013.10.006

[2] Jensen GL Bistrian B Roubenoff R Malnutrition syndromes: a conundrum vs continuum. JPEN J Parenter Enteral Nutr 2009;33:710–6. 19892905 10.1177/0148607109344724

[3] Cederholm T Barazzoni R Austin P ESPEN guidelines on definitions and terminology of clinical nutrition. Clin Nutr 2017;36:49–64. 27642056 10.1016/j.clnu.2016.09.004

[4] Cederholm T Bosaeus I Barazzoni R Diagnostic criteria for malnutrition - An ESPEN Consensus Statement. Clin Nutr 2015;34:335–40. 25799486 10.1016/j.clnu.2015.03.001

[5] White JV Guenter P Jensen G Consensus statement of the Academy of Nutrition and Dietetics/American Society for Parenteral and Enteral Nutrition: characteristics recommended for the identification and documentation of adult malnutrition (undernutrition). J Acad Nutr Diet 2012;112:730–8. 22709779 10.1016/j.jand.2012.03.012

[6] Sorensen J Kondrup J Prokopowicz J EuroOOPS: an international, multicentre study to implement nutritional risk screening and evaluate clinical outcome. Clin Nutr 2008;27:340–9. 18504063 10.1016/j.clnu.2008.03.012

[7] Alvarez-Hernandez J Planas Vila M Leon-Sanz M Prevalence and costs of malnutrition in hospitalized patients; the PREDyCES Study. Nutr Hosp 2012;27:1049–59. 23165541 10.3305/nh.2012.27.4.5986

[8] Lim SL Ong KC Chan YH Malnutrition and its impact on cost of hospitalization, length of stay, readmission and 3-year mortality. Clin Nutr 2012;31:345–50. 22122869 10.1016/j.clnu.2011.11.001

[9] Waitzberg DL Caiaffa WT Correia MI Hospital malnutrition: the Brazilian national survey (IBRANUTRI): a study of 4000 patients. Nutrition 2001;17:573–80. Epub 2001/07/13. PubMed PMID: 11448575. 11448575 10.1016/s0899-9007(01)00573-1

[10] Muscaritoli M Krznaric Z Singer P Effectiveness and efficacy of nutritional therapy: a systematic review following Cochrane methodology. Clin Nutr 2017;36:939–57. 27448948 10.1016/j.clnu.2016.06.022

[11] Cascio BL Logomarsino JV Evaluating the effectiveness of five screening tools used to identify malnutrition risk in hospitalized elderly: a systematic review. Geriatric Nurs (New York, NY) 2018;39:95–102. 10.1016/j.gerinurse.2017.07.006 28943049

[12] Loser C Malnutrition in hospital: the clinical and economic implications. Dtsch Arztebl Int 2010;107:911–7. 21249138 10.3238/arztebl.2010.0911 PMC3023157

[13] Freijer K Tan SS Koopmanschap MA The economic costs of disease related malnutrition. Clin Nutr 2013;32:136–41. 22789931 10.1016/j.clnu.2012.06.009

[14] Montoya Montoya S Munera Garcia NE Effect of early nutritional intervention in the a outcome of patients at risk clinical nutrition. Nutricion Hospitalaria 2014;29:427–36. 24528364 10.3305/nh.2014.29.2.7060

[15] Garcia de Lorenzo A Alvarez J Calvo MV Conclusions of the II SENPE discussion forum on: hospital malnutrition. Nutr Hosp 2005;20:82–7. Epub 2005/04/09. PubMed PMID: 15813390. 15813390

[16] Villalobos Gamez JL Guzman de Damas JM Garcia-Almeida JM Filnut-scale: rationale and use in screening for malnutrition risk within the infornut process. Farm Hosp 2010;34:231–6. 20630782 10.1016/j.farma.2009.12.009

[17] Correia MI Waitzberg DL The impact of malnutrition on morbidity, mortality, length of hospital stay and costs evaluated through a multivariate model analysis. Clin Nutr 2003;22:235–9. Epub 2003/05/27. PubMed PMID: 12765661. 12765661 10.1016/s0261-5614(02)00215-7

[18] Correia M Nutrition screening vs nutrition assessment: what's the difference? Nutr Clin Pract 2018;33:62–72. 28727954 10.1177/0884533617719669

[19] Gordon LJH Compher C Sullivan DG Recognizing malnutrition in adults: definitions and characteristics, screening, assessment and team approach. J Parenter Enter Nutr 2013;37:802–7. 10.1177/0148607113492338 23969411

[20] Schindler K Pernicka E Laviano A How nutritional risk is assessed and managed in European hospitals: a survey of 21,007 patients findings from the 2007–2008 cross-sectional nutritionDay survey. Clin Nutr 2010;29:552–9. 20434820 10.1016/j.clnu.2010.04.001

[21] Anthony PS Nutrition screening tools for hospitalized patients. Nutr Clin Pract 2008;23:373–82. 18682588 10.1177/0884533608321130

[22] Jie B Jiang ZM Nolan MT Impact of nutritional support on clinical outcome in patients at nutritional risk: a multicenter, prospective cohort study in Baltimore and Beijing teaching hospitals. Nutrition (Burbank, Los Angeles County, Calif) 2010;26:1088–93. 10.1016/j.nut.2009.08.027 19963351

[23] Stratton RJ Hackston A Longmore D Malnutrition in hospital outpatients and inpatients: prevalence, concurrent validity and ease of use of the ’malnutrition universal screening tool’ (’MUST’) for adults. Br J Nutr 2004;92:799–808. Epub 2004/11/10. PubMed PMID: 15533269. 15533269 10.1079/bjn20041258

[24] Guigoz Y Vellas B The Mini Nutritional Assessment (MNA) for grading the nutritional state of elderly patients: presentation of the MNA, history and validation. Nestle Nutr Workshop Ser Clin Perform Programme 1999;1:3–11. discussion -2. Epub 2001/08/09. PubMed PMID: 11490593. 10.1159/000062967 11490593

[25] Pilgrim AL Baylis D Jameson KA Measuring appetite with the simplified nutritional appetite questionnaire identifies hospitalised older people at risk of worse health outcomes. J Nutr, Health Aging 2016;20:3–7. 26728926 10.1007/s12603-016-0668-3 PMC4778266

[26] Ferguson M Capra S Bauer J Development of a valid and reliable malnutrition screening tool for adult acute hospital patients. Nutrition 1999;15:458–64. Epub 1999/06/23. PubMed PMID: 10378201. 10378201 10.1016/s0899-9007(99)00084-2

[27] Skipper A Coltman A Tomesko J Position of the academy of nutrition and dietetics: malnutrition (undernutrition) screening tools for all adults. J Academy Nutr Die 2020;120:709–13. 10.1016/j.jand.2019.09.011 31866359

[28] Hirsch S de Obaldia N Petermann M Subjective global assessment of nutritional status: further validation. Nutrition 1991;7:35–7. discussion 7-8. Epub 1991/01/01. PubMed PMID: 1802183. 1802183

[29] Kyle UG Kossovsky MP Karsegard VL Comparison of tools for nutritional assessment and screening at hospital admission: a population study. Clin Nutr 2006;25:409–17. 16356595 10.1016/j.clnu.2005.11.001

[30] van Bokhorst-de van der Schueren MA Guaitoli PR Jansma EP Nutrition screening tools: does one size fit all? A systematic review of screening tools for the hospital setting. Clin Nutr 2014;33:39–58. 23688831 10.1016/j.clnu.2013.04.008

[31] Moher D Shamseer L Clarke M Preferred reporting items for systematic review and meta-analysis protocols (PRISMA-P) 2015 statement. Syst Rev 2015;4:1. 25554246 10.1186/2046-4053-4-1 PMC4320440

[32] Shamseer L Moher D Clarke M Preferred reporting items for systematic review and meta-analysis protocols (PRISMA-P) 2015: elaboration and explanation. BMJ 2015;350:g7647. 25555855 10.1136/bmj.g7647

[33] Moher D Liberati A Tetzlaff J Preferred reporting items for systematic reviews and meta-analyses: the PRISMA statement. J Clin Epidemiol 2009;62:1006–12. 19631508 10.1016/j.jclinepi.2009.06.005

[34] Whiting PF Rutjes AW Westwood ME QUADAS-2: a revised tool for the quality assessment of diagnostic accuracy studies. Ann Intern Med 2011;155:529–36. 22007046 10.7326/0003-4819-155-8-201110180-00009

[35] Landis JR Koch GG The measurement of observer agreement for categorical data. Biometrics 1977;33:159–74. Epub 1977/03/01. PubMed PMID: 843571. 843571

[36] Huedo-Medina TB Sanchez-Meca J Marin-Martinez F Assessing heterogeneity in meta-analysis: q statistic or I2 index? Psychol Methods 2006;11:193–206. 16784338 10.1037/1082-989X.11.2.193

[37] Higgins JP Thompson SG Deeks JJ Measuring inconsistency in meta-analyses. BMJ 2003;327:557–60. 12958120 10.1136/bmj.327.7414.557 PMC192859

[38] Duval S Tweedie R Trim and fill: A simple funnel-plot-based method of testing and adjusting for publication bias in meta-analysis. Biometrics 2000;56:455–63. Epub 2000/07/06. PubMed PMID: 10877304. 10877304 10.1111/j.0006-341x.2000.00455.x

